# DoE Analysis of Approaches for Hydrogel Microbeads’ Preparation by Millifluidic Methods

**DOI:** 10.3390/mi11111007

**Published:** 2020-11-15

**Authors:** Anna Nastruzzi, Gabriele Pitingolo, Giovanni Luca, Claudio Nastruzzi

**Affiliations:** 1 Department of Chemical and Pharmaceutical Sciences, University of Ferrara, 44100 Ferrara, Italy; anna.nastruzzi@edu.unife.it (A.N.); gabriele.pitingolo@bioaster.org (G.P.); 2 Department of Experimental Medicine, University of Perugia, 06123 Perugia, Italy; giovanni.luca@unipg.it

**Keywords:** hydrogel microbeads, millifluidic devices, cell encapsulation, design of experiments, process optimization

## Abstract

Hydrogel microbeads hold great promise for immune-protective cell transplants and in vitro studies. Millifluidic generation of hydrogel microbeads is a highly efficient and reproducible approach enabling a mass production. This paper illustrates the preparation and characterization of highly controlled and reproducible microbeads made by different types of hydrogel using millifluidic approaches. The optimization of the process was made by a design of experiments (DoE) approach. The microbeads’ large-scale production can be potentially used for single cells or clusters encapsulation.

## 1. Introduction

Hydrogel microbeads (HMBs) have emerged for biomedical applications, ranging from drug/cell delivery to the production of scaffolds for tissue engineering and in vitro studies [1].

For instance, extensive research in the application of advanced functional materials for regenerative medicine is focused on the encapsulation of cells and the consequent transplant in a target organ/tissue, providing a cellular replacement and restoring impaired organ functions [2]. The restoration could be accomplished by cells, particularly stem cells, which can perform the repair of appropriate physiologic/metabolic duties, better than chemical-based treatment or mechanical devices [3]. The encapsulation of cells within HMBs plays a key role in cell transplant, isolating the cells from host tissue (providing immune protection) and maintaining the typical phenotype of cells, enhancing the production of biologically active molecules [4]. The possibility to immuno-isolate cells represents indeed a great advance for cell transplantation procedures, overcoming the constraints related to cell sources (i.e., allogeneic or xenogeneic cells), providing alternatives to autologous cells that are often scarcely available [5]. Hydrogel microbeads have been proposed to encapsulate different cell types, including islets of Langerhans, stem cells or inducing pluripotent stem cells, Hepatocytes, Chondrocytes, etc., to mention a few [6,7].

HMBs are typically made of hydrogels able to provide a hydrated environment, which favors the diffusion of low-molecular-weight chemicals essential for cell functionality [8]. Hydrogels consist of hydrophilic polymers that are crosslinked in different ways to form three-dimensional (3D) structures [9]. Once implanted, these highly porous networks elicit scarce protein adsorption as a result of the low interfacial tension with the surrounding fluids [10]. Interestingly, alginate-based HMBs remain almost intact, with the majority of the microcapsules appearing free of fibrotic tissue overgrowth at 8 months after transplant in mice [11]. The production of hydrogel microparticles occurs by mild processes and brings a homogeneous distribution of cells within the network and high biocompatibility due to marginal surface adhesion and protein adsorption in the host.

As a further application, HMBs have been extensively used to build 3D human tissues and organoids for many in vitro assays and drug tests [12]. Microcapsules have been applied to form hepatocyte spheroids with high yield and uniformity for liver tissue engineering [13]. Moreover, organoid cultures in 3D confined matrices are relevant models to faithfully mimic the in vivo environment that supports cell physiological and pathological behaviors [14]. Zhao and colleagues developed a coaxial electrospray system for encapsulation of 3D culture of pluripotent stem cells [15]. More recently, an approach for transgene expression in 3D organoids by combining single-cell encapsulation in Matrigel microbeads, using a microfluidic device and electroporation, was proposed by Laperrousaz and colleagues [16].

In particular, several types of human cells (e.g., stem, red blood or nerve cells, etc.), as well as tumor cells, can be encapsulated in HMBs of predefined dimensions using different methods [14]. The technologies span from batch systems to lithographic and microfluidic approaches. Particularly, micro/millifluidic platforms offer many advantages for cell encapsulation and analysis: not only narrowly dispersed microbeads can be obtained, but also strict control of shape and cell number/bead can be accomplished [17]. Citing a few examples, Rossow et al. presented a droplet microfluidic templating with bio-orthogonal thiol-ene click reactions to prepare cell-laden microgel particles for cell encapsulation [18]. Zheng and colleagues proposed an integrated microfluidic chip to prepare heterogeneous cell-encapsulated alginate microcapsules coupled to mass spectrometry, probing the drug resistance in a tumor-endothelial co-culture model [19]. Although microfluidics offers various advantages, such as the reduction of sample volumes and minute control over the shape and size of microbeads, the quantity of production remains challenging. To solve this drawback, Headen et al. proposed a parallel droplet generator of microgels on a two-layer elastomer device, which has increased throughput vs. single-nozzle devices [20]. Despite the fact that the parallelization of microfluidic chips can increase the number of microencapsulated cells, mass production is still far away. 

The potential of microfluidic-based technology to produce HMBs for medical applications has therefore already been demonstrated. However, the translation of these methods to the industrial environment is still hindered by several limiting factors, mainly related to the relatively small production scale typical of microfluidics. Herein, we propose and optimize millifluidic platforms allowing the formation of highly controlled and reproducible spherical structures, in high yield. The proposed methods are indeed suitable to encapsulate single cells or clusters for large-scale production. 

This paper describes the optimizations by a design of experiments approach (DoE) of two alternative methods for the production of hydrogel microbeads by millifluidics. A detailed overview of the statistical analysis for the two methods is provided including the analysis of the most influencing factors investigated on the response “size”, that represent the mean dimension of the produced HMBs. 

Particularly, different methods and polymers where considered in order to give to the readers a detailed analysis of which factors are important for modifying the dimensional properties of the obtained microbeads suitable for cell encapsulation. A vibrating-nozzle apparatus and a mono air-jet device were selected since they represent valuable examples of equipment for the production of HMBs. 

Also, as proof of concept, the encapsulation of living cells was performed, including an example of tumor cell lines and primary cells.

Notably, this is the first paper describing a comparative analysis of these alternative methods for the production of HMBs also based on different polymers. Moreover, the methods were analyzed by a statistical method in order to establish which factors (experimental parameters) would influence the final dimension of the produced microbeads. We feel this aspect is particularly important to give the reader the possibility to produce microbeads specifically tailored for different cell types in terms of size and formation of 3D microtissues. In addition, the paper describes the use of a less common polymer, represented by the pectin, that could be used in further study aimed to evaluate the in vivo applicability of this polymer.

## 2. Materials and Methods

### 2.1. Materials

Pectin (Unipectine OF 305) was from Degussa Texturant Systems (France) and alginate (Protanal 20/40 e 10/64) was from FMC Biopolymer (Norway). The alginate and pectin dispersions were prepared by dissolving the alginate and pectin powders in bi-distilled water under mechanical stirring. Thereafter, the dispersions were filtered to eliminate the particulate present: the filtration was performed under nitrogen pressure (1.5 bar) using a Millipore filter system (stainless-steel pressure filter holder, 47 mm/340 mL) or Sartorius (stainless-steel filter holder mod. 142 mm/2000 mL) and filter membranes based on glass fiber, cellulose acetate and cellulose nitrate. For the filtration of the hydrocolloid dispersions, glass fiber membranes with a porosity of 5.0, 1.2 and 0.7 μm (Cytivia-Whatman, Kent, UK), and cellulose nitrate with a porosity of 0.45 and 0.22 μm (Merck KGaA, Darmstadt, Germany) were used. The gelling solutions were prepared by dissolving BaCl_2_ in water, under magnetic stirring, and filtered directly with 0.2 μm porosity filters.

### 2.2. Production of Pectin Microbeads by a Vibrating-Nozzle Apparatus

**Figure 1:**
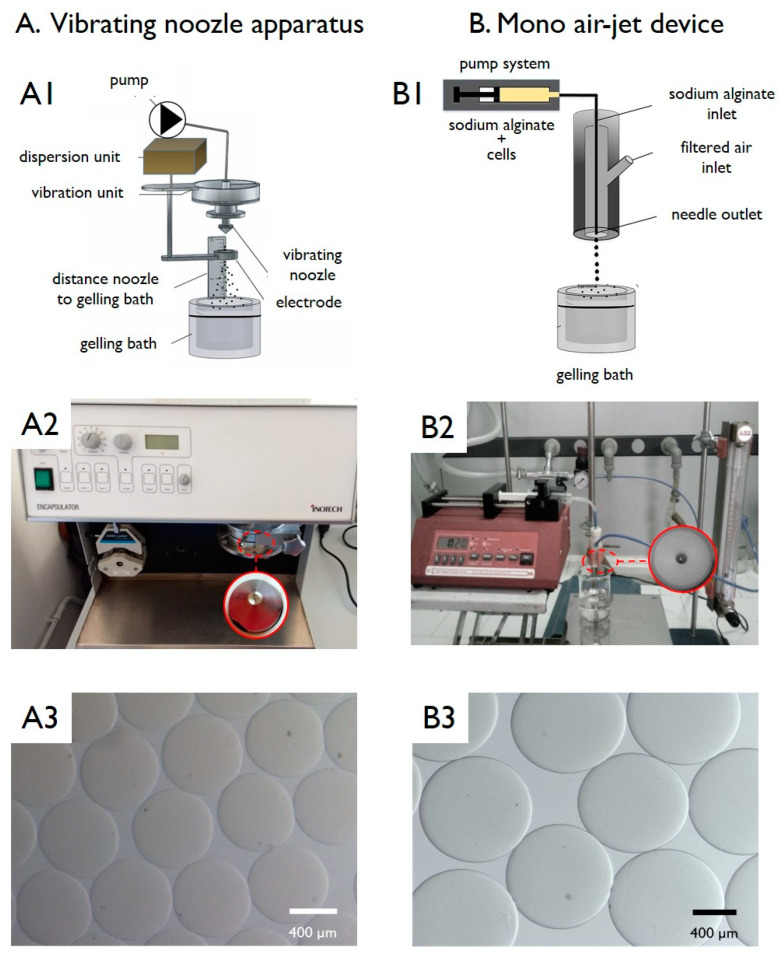
Schematic representation of methods(**A1**,**B1**), pictures of devices (**A2**,**B2**) and optical microphotographs of the empty produced hydrogel microbeads (HMBs) (**A3**,**B3**) by vibrating-nozzle apparatus used to produce pectin microbeads (**A**) and by the mono air-jet device used to produce alginate microbeads (**B**). Scale bars are reported in the panels.

Pectin microbeads were produced using an encapsulation apparatus based on a vibrating-nozzle (Encapsulator Research Inotech, Dottikon, Switzerland). This encapsulator is composed (see Figure 1(A1)) of a glass reaction reservoir with stainless-steel plates: the top plate contains a feed-line connected to a syringe or hydraulic polymer reservoir and a nozzle (300 µm, diameter). The nozzle is connected to a vibrating device, which is insulated from the other components by rubber mounts to avoid the generation of resonance frequencies in the system. The flow of the pectin dispersion in water through the nozzle is achieved by a syringe pump system. Several batches of pectin microbeads were prepared tuning defined experimental parameters by the described design of experiments (DoE) study, followed by statistical analysis. The nozzle vibrational frequency (“freq”), the pectin dispersion pumping rate (“flow”) and the distance between the nozzle and the surface of the gelling bath (“height”) were considered as “factors” (see Table 1). The microdroplets generated by the vibrating-nozzle apparatus were transformed into microbeads by an ionic gelation procedure using a gelling bath constituted by a BaCl_2_ solution (1.5% *w/v*).

### 2.3. Production of Alginate Microbeads by a Mono Air-Jet Device

The alginate microbeads were prepared by a gas-driven mono-jet device [21]. The entire encapsulator system (schematized in Figure 1(B1)) is composed by a gas-driven mono-jet device connected to a precision pump and to a gas flask equipped with a flow meter. Similar to the procedure employed for the preparation of pectin microbeads, different alginate microbead batches were prepared following a DoE approach. The following “factors” were considered: the atomizing gas flow “air”, the alginate dispersion feeding rate “flow” and the distance between the nozzle and the surface of the gelling bath (“height”) (see Table 1). The microdroplets generated by the gas-driven mono-jet device were transformed into microparticles by an ionic gelation procedure using a gelling bath constituted by a BaCl_2_ solution (1.5% *w/v*).

### 2.4. Dimensional and Morphological Characterization of Hydrogel Microbeads

The dimension and morphology of alginate/pectin microbeads were evaluated by optical-stereomicroscopy (Nikon SMZ 1500 stereo microscope, Tokyo, Japan). Microcapsule size and size distribution (by number) were determined by photomicrograph analyses (Eclipsenet version 1.16.5, Laboratory Imaging s.r.o. for Nikon B.V.).

Microbead samples, immediately after preparation and at intervals after storage under different conditions, were applied to a microscope slide and examined microscopically. A sample of around 300 HMBs was examined, and the mean size was determined.

### 2.5. Design of Experiments (DoE)

For studying the influence of different experimental parameters (see details in Table 1) on the morphological characteristics of pectin and alginate microbeads, a randomized central composite face-centered design (CCF) and a Central Composite Circumscribed (CCC) for pectin and alginate microbeads were used, respectively. The parameters were varied as reported in the experimental matrix (see Table 2 and Table 3). The experimental design and the evaluation of the experiments were performed by the PC software MODDE 12.1 (Sartorius Stedim Data Analytics AB, Umeå, Sweden).

### 2.6. Microencapsulation of Cells in Hydrogel Microbeads

As proof of concept, two different cell types were encapsulated in hydrogel microbeads, namely: Sertoli cells (SC) in pectin microbeads and human myeloid leukemia K562 cells in alginate microbeads. Sertoli cells (SC) were encapsulated in pectin microbeads by a vibrating-nozzle apparatus. Briefly, confluent monolayers of SC were scraped off by 0.05% trypsin/EDTA (Gibco, Grandisland, NY, USA) (2 min), washed, counted by hemocytometric analysis, and assayed for viability. Thereafter, SC were suspended in 3% aqueous, highly purified pectin. The pectin SC suspension was continuously aspirated by a peristaltic pump and extruded through the nozzle of the vibrating apparatus with different experimental conditions (see the Results Section) under sterile conditions. Notably, the cell suspension was continuously mixed by a magnetic stirrer to prevent cell clumping, which would possibly lead to inhomogeneous cell distribution within the microcapsules [11]. The generated microdroplets were collected on a BaCl_2_ (1.5% *w*/*v*) gelling bath, whereby they immediately turned into gelled microbeads and were left to incubate for a further 2 min. After the incubation, the obtained microbeads were washed twice in a saline buffer and directly used.

K562 were cultured in Roswell Park Memorial Institute (RPMI) 1640 medium (Gibco, BRL, Milan, Italy) in 10% fetal bovine serum (Gibco, BRL, Milan, Italy) supplemented with 50 units/mL penicillin, 50 µg/mL streptomycin with 5% CO_2_. Before encapsulation, three washing sequences were launched to sterilize all pipelines and liquid passages with a 70% ethanol solution. Alginate microbeads containing K562 cells were prepared using the mono air-jet device; briefly, K562 were suspended in a 1.5% (*w/v*) aqueous alginate at a concentration of 8 × 10^6^ cells/mL. The cell suspension was injected by a syringe pump and extruded through the mono air-jet device, under sterile conditions. The generated microbeads were hardened by an ionotropic gelling process into a 1.5% (*w/v*) BaCl_2_ solution. After 2 min incubation into the gelling bath, the microbeads were washed twice with saline and placed in a medium at 37 °C in a humidified atmosphere of 5% CO_2_. Importantly, to achieve complete biocompatibility, indispensable for further in vitro or in vivo studies, the entire encapsulation procedure was conducted, under sterile conditions (GLP), in a laminar flow hood at room temperature under physiologic pH.

### 2.7. In Vitro Assessment of Encapsulated Cells

After encapsulation, in vitro cultured cells were examined for viability and morphology. Briefly, after different days of culture (after encapsulation), viability was performed by staining the preparations with ethidium bromide (EB) (Sigma-Aldrich, St. Louis, MO, USA) and fluorescein-diacetate (FDA) (Sigma-Aldrich, St. Louis, MO, USA). Cells were visualized under a fluorescence microscope (Nikon, Optiphot-2, Nikon Corporation, Tokyo, Japan) using the filter block for fluorescein. Dead cells were stained in red, while viable ones appeared in green.

## 3. Results and Discussion

### 3.1. Production of Hydrogel Microbeads (HMB)

#### 3.1.1. General Considerations

To allow in vitro or in vivo studies, all the microbeads were prepared in sterile conditions, at room temperature, under physiologic pH using purified and sterile pectin and alginate aqueous dispersions. Notably, the resulting HMBs are transparent, allowing the microscopic observation of cells within the microbead structure (see Section 3.6), facilitating the assessment of cell viability and morphology during in vitro studies. As a gelling ion, barium was chosen (as an alternative of the most commonly employed calcium), since the use of barium ions resulted in the formation of HMBs with extremely high biocompatibility, preserving the in vitro and in vivo viability of the embedded cells, as reported below. In addition, it has been reported that the use of Ba^2+^ ions results in the formation of hydrogels with improved mechanical properties [22]. Importantly, Ba-hydrogel devices can be transplanted in vivo without the need for a further coating. Barium has sometimes been considered a toxic chemical and concerns have been raised about the use of Ba^2+^ ions as ionic crosslinkers. Published studies have indeed demonstrated that the use of low barium concentrations and a short gelling time resulted in the production of very safe alginate devices without toxic effects [23].

#### 3.1.2. Experimental Design

It is well known that the operational parameters can strongly affect the production of HMBs and in particular their dimensional and morphological characteristics. Therefore, in the present paper, the statistical optimization of the operational conditions was studied in detail by an analysis based on the “design of the experiments” (DoE) approach. Nevertheless, before undertaking the DoE, a preliminary classical intuitive approach “COST” (Changing One Separate factor a Time), was performed to select the important factors and their range of variation. Table 1 summarizes the entire set of the investigated experimental parameters, including abbreviation, meaning and range of variation.

In order to also have further and comparative results on different techniques and polymers, the production of HMBs was performed using two methods and two polymers, namely: a vibrating-nozzle apparatus, for the production of pectin HMBs, and a mono air-jet device, for alginate HMBs. The general procedure for both approaches is schematized in Figure 1(A1,B1), as well photographs of the platforms (Figure 1(A2,B2)) together with the optical microphotographs of both pectin (Figure 1(A3)) and alginate (Figure 1(B3)) empty microbeads.

#### 3.1.3. Microbeads Formation

Irrespectively of the preparation strategy, the microcapsule formation consists of three main steps, as described below:

1. The preparation of the aqueous colloidal polymeric dispersion (the polymer is typically employed at a concentration ranging from 1% to 3%, *w*/*v*).
2. The second step is represented by the formation of the *sol* polymer droplets using a highly controllable strategy, aimed at the formation of dimensionally homogeneous microdroplets. This step is the critical phase of the process: droplets are generally obtained by forcing the liquid polymeric dispersion through a nozzle/needle, causing the formation of the droplets by different mechanisms.
  Specifically, in the experiments described in the current paper, the liquid polymeric dispersion generates a laminar jet that breaks into single droplets by the vibration of the micronozzle (in the case of pectin) and an air-jet (in the case of alginate).
3. The final step provides for the hardening of the generated droplets by a gelation procedure that causes the formation of the HMBs.
  Specifically, the formation of uniform polymeric microdroplets (step 2) represents the most critical one of many preparation processes described in the literature. The underlying principle of the droplet generation is that a liquid, when forced through a nozzle, is extruded initially as individual droplets. By varying different experimental settings such as the nozzle diameter, the pumping rate and the applied air-flow or electric field, the droplet diameter can be adjusted.

#### 3.1.4. Dimensional Considerations

Finally, dimensional consideration was made. Since microbeads are designed for cell encapsulation and considering that the size of cells or cell clusters usually exceeds one hundred µm, microbeads with a mean diameter above 500–600 up to 1200 µm (for cell clusters) are desired. If the microbead diameter would be too small (i.e., <400 µm), the number of partially protruding cells could be proportionally important, causing an increase of negative inflammatory responses after in vivo transplantation. Consequently, microbead production methods were tailored with a target size comprised between ~700 and 1200 µm.

Concerning the barium concentration in the gelling batch, the choice of employing BaCl_2_ at a concentration of 1.5% (*w/v*) was made. This particular concentration was selected based on many previous studies, showing that the structural and mechanical properties of ionically cross-linked hydrogels largely depend on the ionic strength of the gelation medium and the ion source [24].

### 3.2. Design of Experiments (DoE)

In general terms, the design of experiments (DoE) statistical approach can be considered as a way of choosing examples in the space of design, to obtain the maximum information using minimum resources.

DoE offers indeed a rational approach for experiment-based research by reducing the number of experiments and providing information about the effects of different variables and their interactions. In addition, the DoE process can help to improve the accuracy with which it is possible to predict the operating characteristics of many production procedures.

In this study, DoE analysis was employed to study the optimization of HMBs production. Three experimental parameters were selected as key factors for each production approach and two slightly different statistical models were employed.

Namely, a Central Composite Face design (CCF) for the analysis of pectin microbeads production, which is composed of a fractional factorial design and star points placed on the faces of the sides. Whereas, a Central Composite Circumscribed design (CCC) was selected to study alginate microbeads fabrication, in this case, the DoE is composed of a fractional factorial design and star points (star distance equal to 1.68).

The two designs, CCF and CCC, were chosen to optimize the result by the Response Surfaces Method (RSM). RSM refers to a set of mathematical and statistical techniques useful in the modeling and analysis of problems in which a response of interest is influenced by different variables and the objective is to optimize the response. 

In most RSM problems, the form of the relationship between the answer and the independent variables is unknown. The first step is therefore to find a suitable one approximation of the true functional relationship between Y (response) and the set of variables independent (factors) through multiple linear regression theory (MRL). Usually, a low-order polynomial model is used in a predetermined region of the independent variables.

### 3.3. Production of Pectin Microbeads by a Vibrating-Nozzle Apparatus

**Figure 2:**
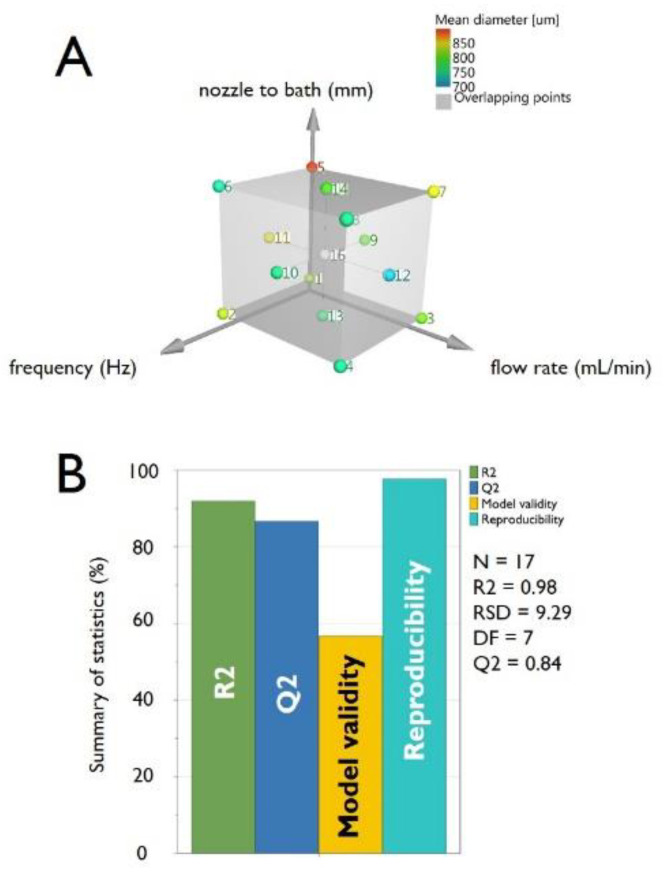
Panel (**A**) scheme of the Central Composite Face-Centered Design (CCF) used for the DoE relative to the production of pectin microbeads by the vibrating-nozzle apparatus. Panel (**B**) histogram showing the summary of statistics for pectin microbeads production. The bars represent the four main parameters, namely: R2, Q2, model validity and Reproducibility. R2 represents the coefficient of determination (also sometimes called the multiple correlation coefficient) while Q2 estimates the predictive ability of the model.

**Figure 3:**
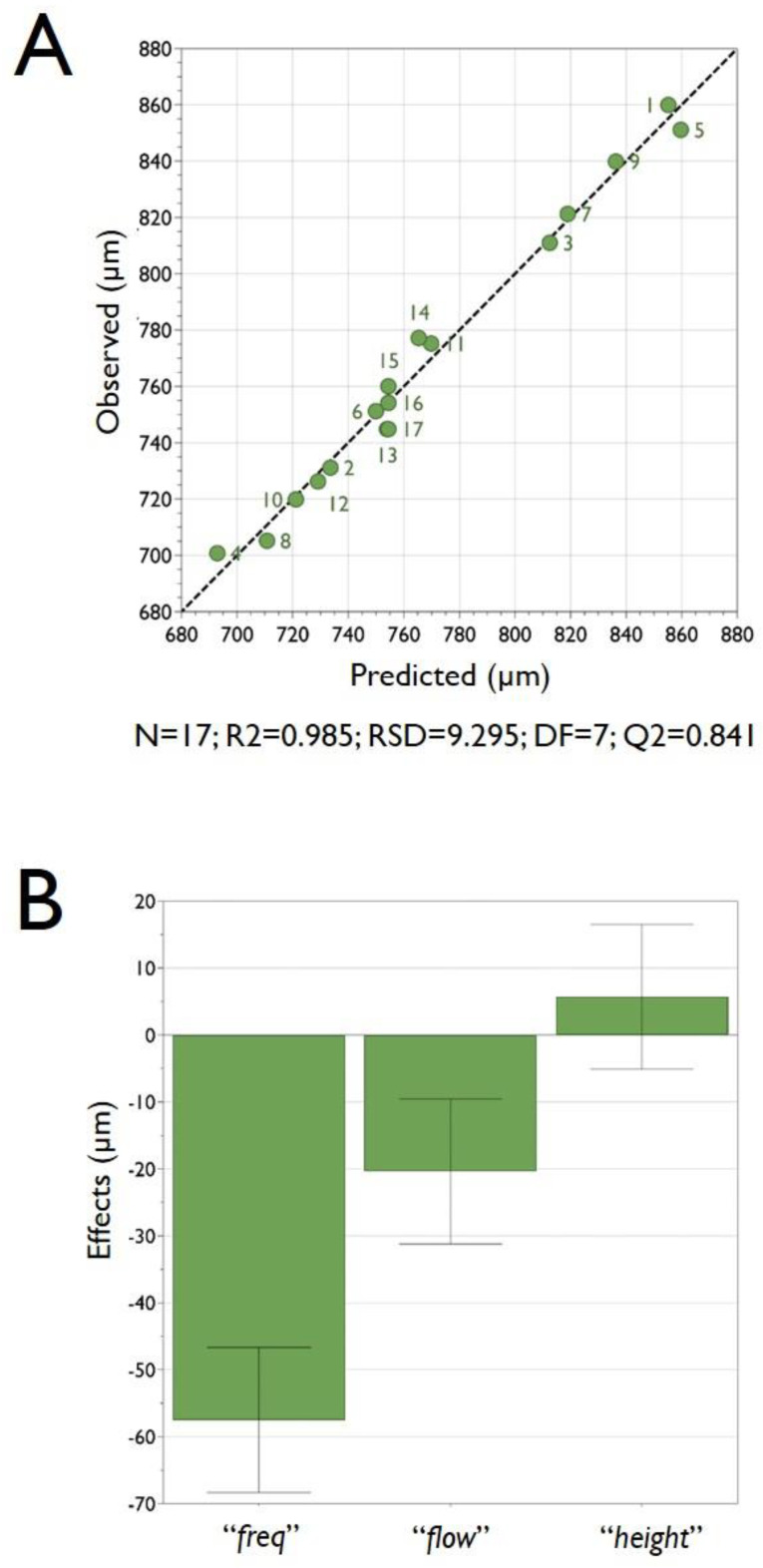
Panel (**A**) the graph reports the observed vs predicted plot for pectin microbeads production. The data points reported in the figure represent the average dimensions of the produced microbeads. The determination of the microbeads size was determined by optical microphotograph analysis of around 300 microbeads, as reported in the experimental section. The numbers reported in the figure close to each data point refer to the experiments performed following the experimental plan required for the DoE analysis as reported in Table 2. Panel (**B**) the coefficient plot presents a graphical representation of the terms “*freq*”, “*flow*” and “*height*” for pectin microbeads production. The reported values help in determining the significance of the factors.

**Figure 4:**
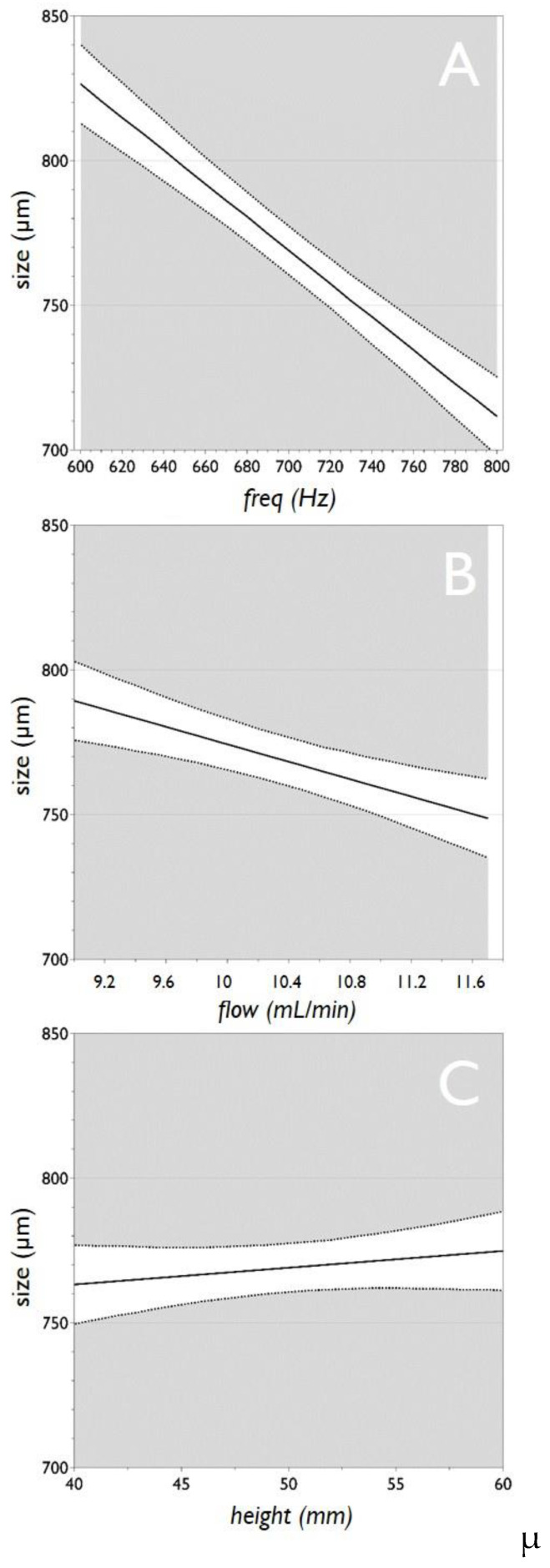
Main effect plots for the factors “*freq*” (**A**), “*flow*” (**B**) and “*height*” (**C**), versus the response “*size*” for the preparation of pectin microbeads. Graphs report the predicted values of the response when the factors vary from its low to its high level. Dotted lines represent the lower and upper limits of a confidence interval.

**Figure 5:**
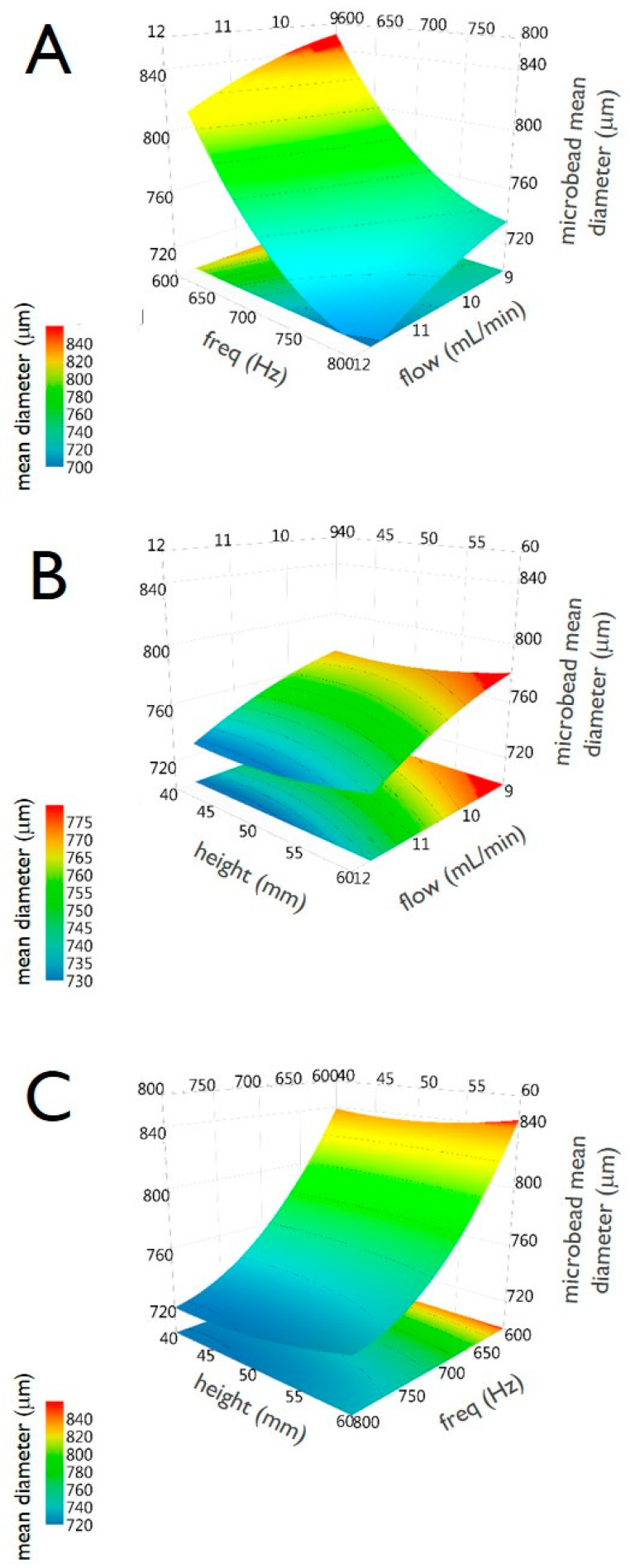
Response surface plots of the response “*size*” for the interaction between “*freq*” and “*flow*” (**A**), between “*height*” and “*flow*” (**B**) and between “*height*” and “*freq*” (**C**). Data are relative to the preparation of pectin microbeads.

The encapsulation procedure based on the use of the vibrating-nozzle apparatus (schematized in Figure 1A), represents a useful approach for the production of polysaccharide microbeads, intended for cell encapsulation. The DoE analysis relative to pectin HMBs is described in Figure 2, Figure 3, Figure 4 and Figure 5.

The factors taken into consideration are: the vibrational frequency (“*freq*”), which is the frequency of vibration applied to the nozzle (measured in Hz), the flow rate (“*flow*”), which is the pumping rate of the feeding pectin dispersion (measured in mL/min), and the distance nozzle to gelling bath (“*height*”), which is the distance between the nozzle tip and the surface of the barium ion containing gelling bath (measured in mm). Each factor is varied on three levels: −1/0/+1 (defined as low, middle and high levels, respectively).

The response variable to be optimized is the average size (µm) of the HMPs obtained (“*size*”).

In consideration of the factors and the levels chosen, starting from a 3^3^ plane, we move to an experimental plan 2^3^ + 2 × 3 + 1 (a Central Composite Face-Centered Design, CCF), schematized in Figure 2A. The design is composed of a fractional factorial design (2^3^), star points (2 × 3) and replicated center points (1). Performing one repetition for the points on the cube and in the axial points and 3 repetitions in the central point, a total of 8 + 6 + 3 = 17 experiments have been therefore conducted (as summarized in Table 2).

The summary of statistics is summarized in Figure 2B and presents four parameters (R2, Q2, Model validity and Reproducibility). R2 represents the model fit (i.e., a R2 value lower than 0.5 is a model with rather low significance). In our case, we obtained the optimal value of R2 = 0.985. Also, Q2, representing the estimation of the future prediction precision (i.e., the best and most sensitive indicator), is very good at 0.84. Q2 should be indeed greater than 0.1 for a significant model and greater than 0.5 for a good model. The difference between R2 and Q2 should also be smaller than 0.3 for a good model, being, in our experiments, 0.14.

Model validity is a test of diverse model problems. A value less than 0.25 for model validity indicates statistically significant model problems, such as the presence of outliers, an incorrect model, or a transformation problem. In the set of experiments, model validity resulted to be 0.77, representing again a very good value, reflecting a high validity. Finally, reproducibility, representing the variation of the replicates compared to overall variability, was remarkably good with a value of 0.98 (the reproducibility should be greater than 0.5).

As a further analysis of the DoE experiments, Figure 3A reports the observed vs predicted plot; as clearly appreciable from the graph, the point distribution that is very close to a straight line indicates a good model. The R2 value of 0.98 clearly confirms the great accuracy of the model, and there is indeed a strong correlation between the model’s predictions and its actual results.

The coefficients plot, reported in Figure 3B, presents a graphical representation of the model factors, in order to assess their significance. In fact, when a factor has a value far away from y = 0 (either positive or negative), it indicates a strong significance over the analyzed response. Also, when the uncertainty range bars of a specific factor do not cross y = 0, it indicates that such a factor is highly significant. In this respect, from the analysis of the plot reported in Figure 3B, it is evident that the most significant factor (i.e., that one is more pronounced affecting the mean size of HMBs) is the factor “*freq*” followed by “*flow*”, whereas the factor “*height*” had only a very marginal significance over the investigated response “*size*” (that represents the mean dimension of the produced HMBs). The factor “*freq*” has an important role since the frequency of the vibration of the nozzle deeply influences the dimension of the droplets that are later converted into microbeads. Please note that the reported coefficient plot displays the coefficients, when changing from the middle to high, for the selected response with the confidence interval as error bars. By default, the coefficients refer to the data scaled and centered.

The main effect plots are reported in Figure 4. For each of the three analyzed factors, the plot displays the predicted values of the response “*size*” when the factors vary over the range (i.e., from low to high), all other factors in the design are held constant at their middle value. It is important to note that when the line is horizontal (parallel to the x-axis), there is no main effect present. The response mean is the same across all factor levels. On the other hand, if the line is not horizontal, there is a main effect present. The response mean is not the same across all factor levels. Therefore, taking into consideration what is stated above, the plots of Figure 4 confirm that the factor “*freq*” has a major effect on “*size*”, followed by “*flow*”, whereas the factor “*height*” has only a marginal effect on the microbead size.

Finally, Figure 5 includes the three surface response plots. They display the predicted “*size*” values, spanned by two factors, in a response surface plot. Specifically, panel A reports “*size*” spanned by “*freq*” and “*flow*”, panel B by “*height*” and “*flow*” and panel C by ”*height*” and “*freq*”.

### 3.4. Production of Alginate Microbeads by a Mono Air-Jet Device

Analogously to the experiments run for the pectin microbead, alginate beads production was analyzed by a DoE approach with some modifications. Firstly, in the case of alginate, a tailor-made encapsulator was employed. In our laboratory, we have indeed designed and fabricated a new model of coaxial bead generator, named a “gas-driven mono-jet device”. The entire project was developed to improve some instrumental characteristics and performances of coaxial bead generators available on the market, including the connectivity (to alginate-cell suspension and gas/air generator) and the feature to allow the change of the internal diameter of the nozzle, this allows the production of microcapsules of different sizes.

In this respect, our device is equipped with two lateral and one top standard rapid connectors based on female luer lock (as air and alginate inlets) and an internal nozzle (commercially available blunt-end needles) that is easily interchangeable, depending on the dimensions required. The generated microdroplets are then consolidated by a barium ion gelling bath. Typically, the cell suspension is continuously mixed by a magnetic stirrer to prevent cell clumping, which could lead to inhomogeneous cell distribution within the microparticles [11].

The second variation (i.e., for the pectin experiments) relies on the DoE protocol adopted. In consideration of the factors and the levels chosen, starting from a 3^3^ plane, we move to an experimental plan 2^3^ + 2 × 3 + 1 (CCC = Central Composite Circumscribed) chosen for optimizing the result (RSM response surfaces method). This design is composed of a 2^3^ factorial design: number of points on the cube, plus star points (axial) 2 × 3: number of axial points outside the cube determined through a preliminary screening, and finally, of the repeated central point. Performing one repetition for the points on the cube and in the axial points and 3 repetitions in the central point, a total of 8 + 6 + 3 = 17 tests have been conducted. This factorial plan was chosen because the CCC design provides higher forecast quality over the entire design space, however, it requires factor settings outside the range of factors in the factorial part (requires 5 levels for each factor). The value out of the plane is chosen for the ability of the model to provide good forecasts throughout the region of interest (equal estimation accuracy in all directions). In this case, therefore, we have α = (2*^k^*)^1/4^ = (2^3^)^1/4^ = 1.682.

The factors taken into consideration were, again (see Table 1): flow with which the alginate is fed (“*flow*”), atomizing airflow (“*air*”), the distance between the nebulizer nozzle, and the free surface of the gelling bath (”*height*”). By definition of the CCC design, each factor was, therefore, varied at five levels: −1.68/−1/0/+1/+1.68, the response variable to be optimized was, as for pectin microbeads, the average size of the microparticles obtained (“*size*”).

**Figure 6:**
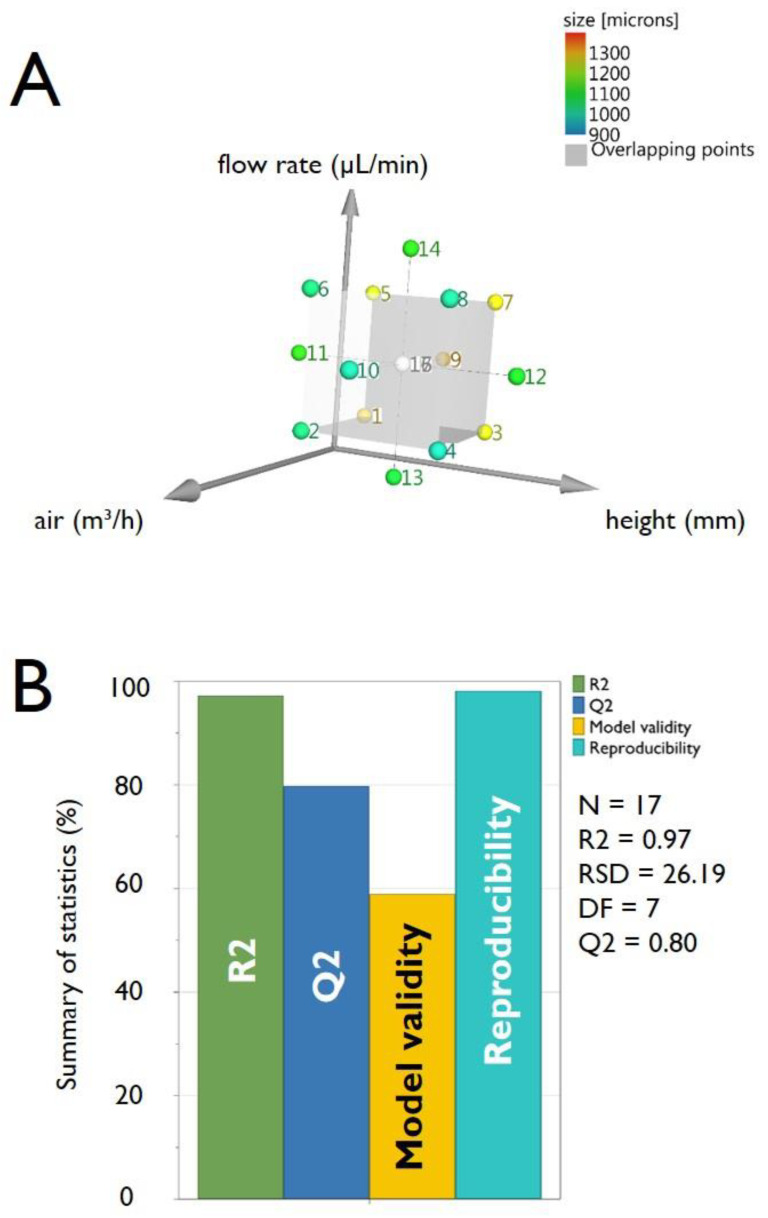
Panel (**A**) scheme of the Central Composite Circumscribed Design (CCC) used for the DoE relative to the production of alginate microbeads by the mono air-jet device. Panel (**B**) histogram showing the summary of statistics for alginate microbeads production. The bars represent the four main parameters, namely: R2, Q2, model validity and Reproducibility. R2 represents the coefficient of determination (also sometimes called the multiple correlation coefficient) while Q2 estimates the predictive ability of the model.

**Figure 7:**
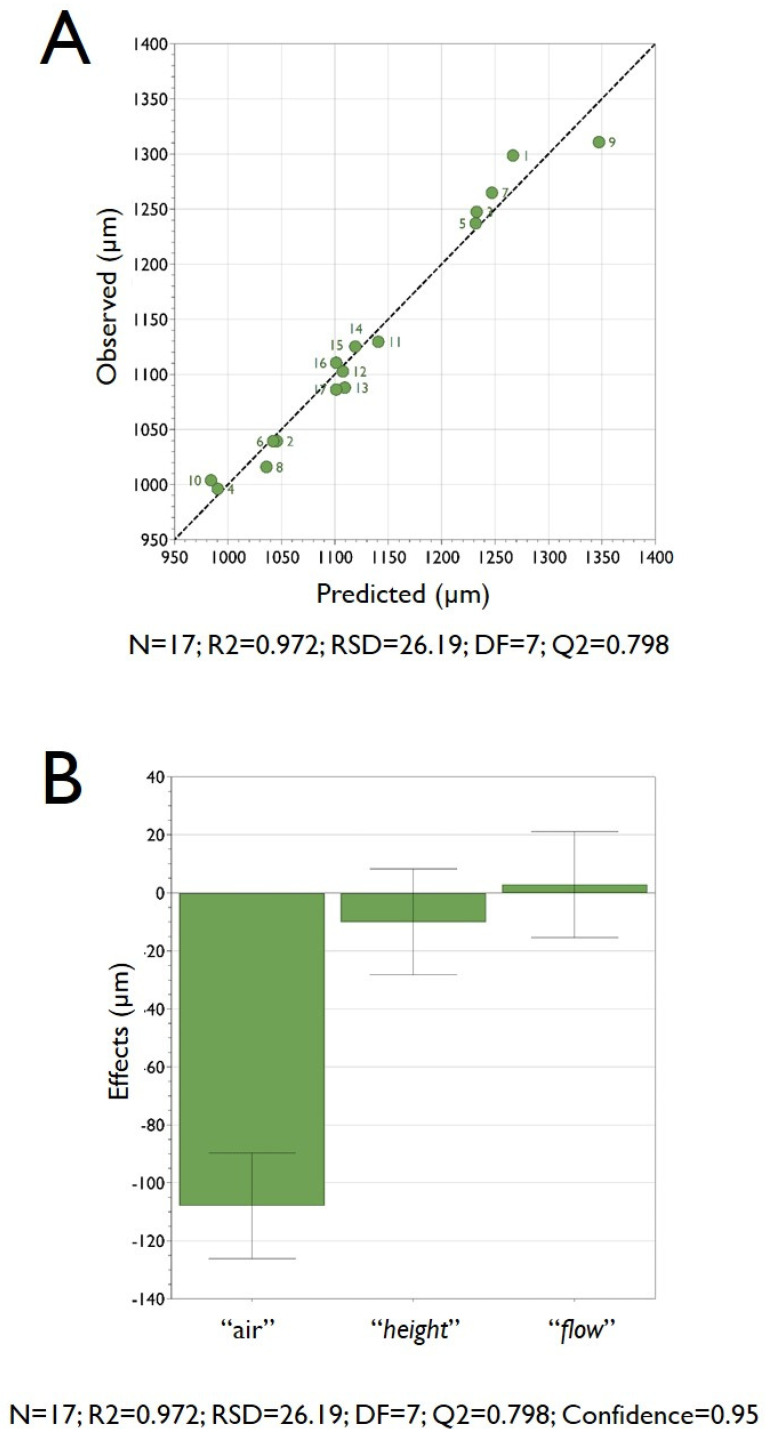
Panel (**A**) the graph reports the observed vs predicted plot for alginate microbeads production. The data points reported in the figure represent the average dimensions of the produced microbeads. The determination of the microbeads size was determined by optical microphotograph analysis of around 300 microbeads, as reported in the Experimental Section. The numbers reported in the figure close to each data point refer to the experiments performed following the experimental plan required for the DoE analysis, as reported in Table 3. Panel (**B**) the coefficient plot presents a graphical representation of the terms “*air*”, “*height*” and “*flow*” for alginate microbeads production. The reported values help in determining the significance of the factors.

**Figure 8:**
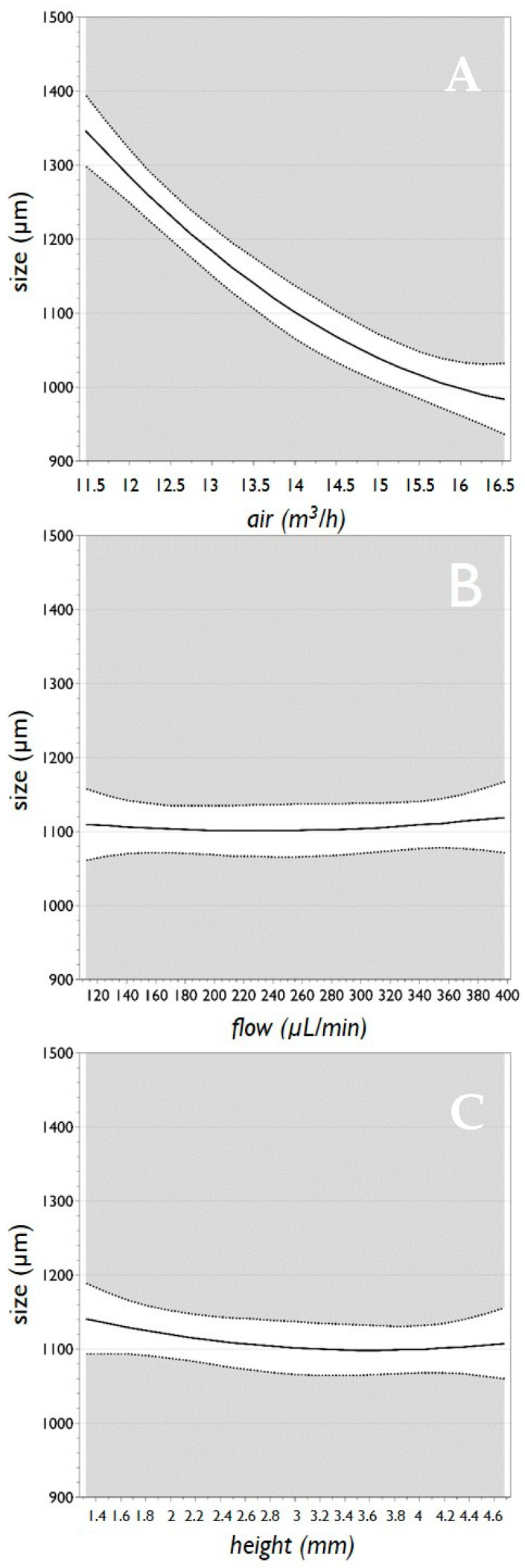
Main effect plots for the factors “*air*” (**A**), “*flow*” (**B**) and “*height*” (**C**), versus the response “*size*” for the preparation of alginate microbeads. Graphs report the predicted values of the response, when the factors vary from its low to its high level. Dotted lines represent the lower and upper limits of the confidence interval.

**Figure 9:**
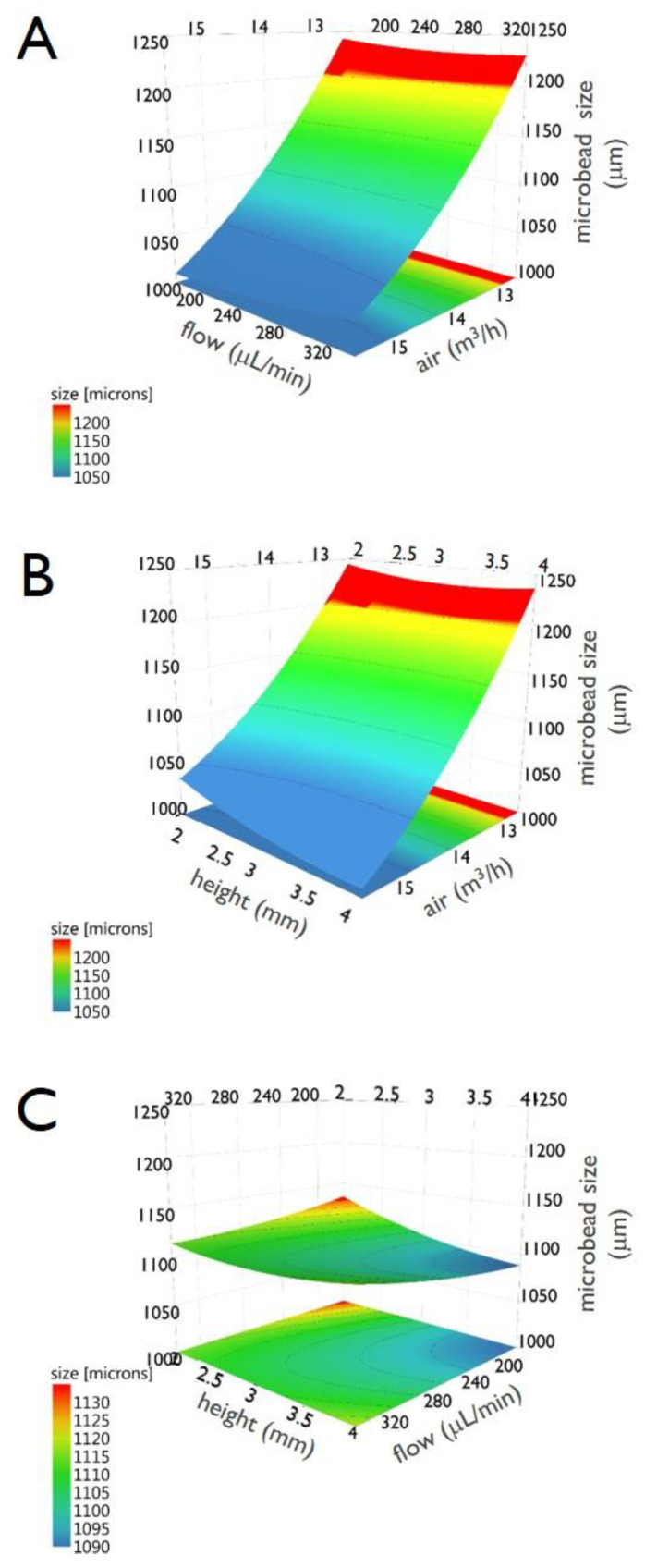
Response surface plots of the response “*size*” for the interaction between “*flow*” and “*air*” (**A**), between “*height*” and “*air*” (**B**) and between “*height*” and “*flow*” (**C**). Data are relative to the preparation of alginate microbeads.

As thoroughly described for the DoE analysis of pectin HMBs production, Figure 6, Figure 7, Figure 8 and Figure 9 report the following information relative to the preparation of alginate HMBs: the experimental plan (Figure 6A), the summary of statistics (Figure 6B), the observed vs predicted plot (Figure 7A), the coefficients plot (Figure 7B), the main effect plots (Figure 8) and the surface responses plots (Figure 9).

As described for pectin HMBs, the summary of statistics, reported in Figure 6B, indicates the optimal validity of the design performed, as proved by the very high value of R2 = 0.97, Q2 at 0.80 and a difference between R2 and Q2 being, in our experiments, very low, at 0.17. Moreover, in the set of experiments carried out for alginate HMBs, model validity resulted to be 0.59, representing again a very good value, reflecting a high validity. Finally, reproducibility, representing the variation of the replicates compared to overall variability, was remarkably good, with a value of 0.98 (the reproducibility should be greater than 0.5).

Notably, the analysis of 3D surfaces plots (reported in Figure 9), clearly indicates that a marked modification (i.e., reduction) in the diameter of the HMBs is obtained for high “*air*” for all “*flow*” values (at constant “*height*”), for high “*air*” for all “*height*” values (at constant “*flow*”) and for “*height*” set high and “*flow*” set low (at constant “*air*”).

### 3.5. Encapsulation of Cells in Hydrogel Microbeads

The possibility to immuno-isolate cells represents a significative improvement for cell transplantation in clinics; specifically, it allows to overcome constraints related to cell sources (i.e., allogenic or xenogenic cells), providing alternatives to autologous cells that are often scarcely available. Therefore, cell-based medicines require the use of an embedding scaffold, forming a biocompatible shell that, once transplanted, provides the immuno-isolation of the cells, isolating them from the host immune system, without requiring the administration of immunosuppressive drugs.

In this respect, the biomaterial and the geometrical and morphological aspects of the encapsulating device play a fundamental role in cell transplantation procedures. Notably, the encapsulation of cells in HMBs provides precise control over proliferation and cell morphology in a tailored geometric 3D structure.

Special regard was given to the study of morphological and dimensional characteristics of the microbeads with the specific aim to entrap living cells, possibly avoiding the frequently encountered morphological problems represented by the presence of coalescences, irregular tail-shaped microbeads, cracks or a rough/waved and wrinkled surface.

It is indeed very important to study in detail the HMBs production process to avoid any microbead defects, since they can cause a severe immuno-response. After implantation, beads’ irregularities are immediately covered by plasma proteins, activating the subsequent cell adhesion.

**Figure 10:**
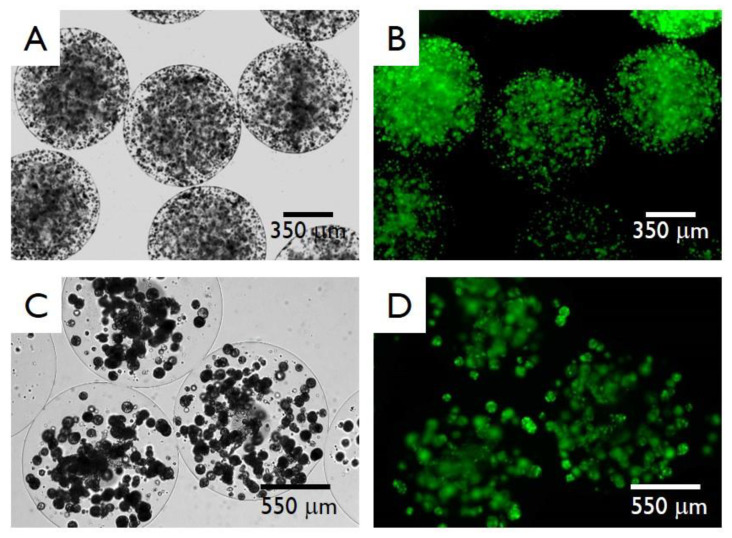
Representative optical (**A**,**C**) and fluorescence (**B**,**D**) microphotographs of pectin microbeads (**A**,**B**) and alginate microbeads (**C**,**D**). Pectin and alginate beads respectively encapsulate Sertoli Cells and K562 cells. Fluorescence viability tests were performed after 48 h from the encapsulation. For the viability test, the HMBs were produced with the central point conditions (see experiments 15, 16 and 17 in Table 2 and Table 3). Scale bars are reported in the panels.

In consideration of what is stated above, we have therefore produced both pectin and alginate HMBs respectively, encapsulating SC and K562 cells. Optical and fluorescence microscopic examination of the microbeads produced with both pectin (Figure 10A,B) and alginate (Figure 10C,D) demonstrate that the HMBs produced with the optimized experimental parameters were spherical in shape and characterized by a smooth surface with the absence of main morphological defects (such as coalescences or tails). Moreover, the fluorescence photomicrographs reported in Figure 10B,D shows the great viability of the encapsulated cells, after 48 h of cell culture, demonstrating that the cells were highly viable after the encapsulation. Notably, cells were visualized under a fluorescent microscope: dead cells were stained in red, while viable cells appear in green. As clearly appreciable from the reported microphotographs, the number of red cells is almost undetectable and the cell viability was very high, confirming our previous results [24].

### 3.6. Concluding Comments

Encapsulating mammalian cells in hydrogel-based scaffolds, in the form of microbeads, is gaining popularity. The success of this technology is mainly due to the possibility of producing scaffolds with precise morphological and functional properties for a number of applications, including in vitro cell proliferation tests and cell-based therapy. For the use of optimized and reproducible protocols, it is mandatory to obtain better control over the dimensions and morphology, together with the possibility to combine, in a single process, scaffold fabrication and cell embedding. In the current paper, we have indeed demonstrated that by changing the production parameters (typified by the analyzed factors), it is possible to tailor the dimensional characteristic of the HMBS. It is indeed important to underline that a single set-up for HMBs preparation suitable for all cell types does not exist; on the contrary, depending on the dimensions of cells and their aggregation state (e.g., the formation of spheroids or 3D microtissues), the dimensions of HMBs can be modified by adjusting the preparation factors. 

With respect to the commercial applicability of cell-based medicines, further studies are in due course in our laboratory to exploit the possibility to pass from the lab-scale equipment presented in the current paper towards the development of protocols suitable for the pilot-scale level.

## Figures and Tables

**Table 1:** List of the investigated experimental parameters, including abbreviation, meaning and range of variation.

Production of Pectin Microbeads by a Vibrating-Nozzle Apparatus			
Experimental Parameter (Factor)	Abbreviation	Meaning	Range of Variation
Vibrational frequency	“*freq*”	Frequency of vibration applied to the nozzle (Hz)	600–800
Flow rate	“*flow*”	Pumping rate of the feeding pectin dispersion (mL/min)	9–11.7
Distance nozzle to gelling bath	“*height*”	Distance between the nozzle tip and the surface of the barium ion containing gelling bath (mm)	40–60
**Production of alginate microbeads by a mono air-jet device**			
Atomizing air	“*air*”	Flow of the atomizing air pumped into the jet device (m^3^/h)	11.5–16.5
Distance needle to gelling bath	“*height*”	Distance between the needle outlet and the surface of the barium ion containing gelling bath (mm)	13.2–46.8
Flow rate	“*flow*”	Pumping rate of the feeding alginate dispersion (µL/min)	112.0–398.0

**Table 2:** Summary of design of experiments (DoE) performed for the optimization of pectin microbead production by a vibrating-nozzle apparatus.

Experiment No	Run Order	Freq (Hz)	Flow (mL/min)	Height (mm)	Size (µm)
1	6	600	9.0	40	860
2	8	800	9.0	40	731
3	7	600	11.7	40	811
4	13	800	11.7	40	701
5	5	600	9.0	60	851
6	1	800	9.0	60	751
7	16	600	11.7	60	821
8	2	800	11.7	60	705
9	15	600	10.3	50	840
10	10	800	10.3	50	720
11	11	700	9.0	50	775
12	12	700	11.7	50	726
13	17	700	10.3	40	745
14	9	700	10.3	60	777
15	14	700	10.3	50	760
16	3	700	10.3	50	754
17	4	700	10.3	50	745

**Table 3:** Summary of DoE performed for the optimization of alginate microbead production by a mono air-jet device.

Experiment No	Run Order	Air (m^3^/h)	Height (mm)	Flow rate (µL/min)	Size (µm)
1	7	12.5	20.0	170	1298
2	9	15.5	20.0	170	1039
3	5	12.5	40.0	170	1247
4	10	15.5	40.0	170	995
5	2	12.5	20.0	340	1237
6	12	15.5	20.0	340	1039
7	15	12.5	40.0	340	1264
8	6	15.5	40.0	340	1015
9	3	11.5	30.0	255	1311
10	17	16.5	30.0	255	1003
11	11	14.0	13.2	255	1129
12	8	14.0	46.8	255	1102
13	13	14.0	30.0	112	1087
14	14	14.0	30.0	398	1125
15	1	14.0	30.0	255	1110
16	4	14.0	30.0	255	1110
17	16	14.0	30.0	255	1085
